# The Persistence of Oral Health Disparities for African American Children: A Scoping Review

**DOI:** 10.3390/ijerph16050710

**Published:** 2019-02-27

**Authors:** Dominique H. Como, Leah I. Stein Duker, José C. Polido, Sharon A. Cermak

**Affiliations:** 1 Mrs. T.H. Chan Division of Occupational Science and Occupational Therapy, Herman Ostrow School of Dentistry, University of Southern California, Los Angeles, CA 90089, USA; lstein@chan.usc.edu (L.I.S.D.); cermak@chan.usc.edu (S.A.C.); 2 Children’s Hospital Los Angeles and Herman Ostrow School of Dentistry, University of Southern California, Los Angeles, CA 90089, USA; JPolido@chla.usc.edu

**Keywords:** dental care, oral care, minority, disparity, African American, social determinants of health

## Abstract

Oral health is an important yet often neglected component of overall health, linked to heart disease, stroke, and diabetic complications. Disparities exist for many groups, including racial and ethnic minorities such as African Americans. The purpose of this study was to examine the potential factors that perpetuate oral health care disparities in African American children in the United States. A systematic search of three literature databases produced 795 articles; 23 articles were included in the final review. Articles were analyzed using a template coding approach based on the social ecological model. The review identified structural, sociocultural, and familial factors that impact the ability of African Americans to utilize oral care services, highlighting the importance of the parent/caregiver role and the patient–provider relationship; policy-level processes that impact access to quality care; the value of autonomy in treatment and prevention options; and the impact of sociocultural factors on food choices (e.g., food deserts, gestures of affection). In conclusion, oral health care remains an underutilized service by African American children, despite increasing access to oral care secondary to improvements in insurance coverage and community-based programs.

## 1. Introduction

Oral health is an important yet often neglected component of overall health. According to the Surgeon General’s report, “Oral Health in America”—the first report of its kind to focus on oral health needs exclusively—there is “*a silent epidemic of dental and oral diseases … a burden of diseases that restricts activities...”* [1] (p. 17). This report served as a catalyst for several policy changes related to oral health care over the last decade. However, Surgeon General Vivek Murthy (2014–2017) noted that while advances have been made, underserved and vulnerable populations continue to suffer from the effects of poor oral health at disproportionate rates [2]. As such, oral health continues to be a priority focus area in Healthy People 2020, with the goal to improve oral health by increasing access to dental care services and minimizing oral and craniofacial diseases, conditions, and injuries through prevention and treatment [3]. Beyond being an indicator of well-being, the overarching value of good oral health can be found in its capacity to improve a person’s ability to engage with others, express emotions and allow for the more fundamental functions of smell, taste, touch, chew, and swallow [3].

Conversely, poor oral health has contributed to lost productivity for both children and adults. Children who experience dental pain or untreated infections are more likely to perform poorly in school or miss it altogether. As a result of oral health problems, children miss upwards of 50 million school hours, which translates to 10 million school days [4]. Increased absenteeism rates from school for the child often result in missed work days for their caregivers [5,6]. Therefore, it is no surprise that many health promotion policies, campaigns, and interventions have attempted to target oral health.

Improving access to oral health services for pregnant women and children has been the primary aim of previously implemented, ratified, and/or funded public health policies since the publication of the 2000 Surgeon General’s report. For example, funding of preventative services has increased for Medicaid coverage plans whereby dental benefits must be included by any state that provides Children’s Health Insurance Program coverage through a Medicaid expansion program [7]. Also, several other low-cost discount plans have come to the marketplace as an alternative or supplement to other forms of coverage.

Public health agencies have also attempted to increase access to services by situating programs within the community, such as school-based dental sealant programs in which a dental professional applies a sealant to the teeth to protect against cavities [1]. These programs are typically housed in communities with a large number of vulnerable children at greater risk for developing caries, thereby increasing the reach of dental practitioners to the community, as well as acting as a cost-saving measure by reducing the need for costly emergency dental treatments [8]. Another program, community-based water fluoridation, characterized by the addition of fluoride to the public water system, has been touted as one of the major advancements in evidence-based strategies to combat tooth decay [9]. According to the CDC [8], fluoridation is responsible for decreasing one-quarter of caries in adults and children. Several organizations support the use of fluoride and community-based fluoridation, including the World Health Organization, which has officially endorsed the use of fluoride in water, salt, and milk for population-based prevention of dental cavities since the late 1960s [10].

Despite these policies, campaigns, and interventions targeting oral care, health disparities exist for minority children (e.g., African American, Native American, and Latino) compared to Caucasian children [11]. For African Americans in particular, oral health remains a profound problem. African American children and adolescents had higher levels of dental caries and in 2009–2010, the prevalence of untreated caries for African American children aged 3–5 was significantly higher than Caucasian children (19% vs. 11%, respectively) [12]. This gap widened when children reached adolescence; by 13–15 years old, the prevalence of untreated caries for African American adolescents were almost three times as high (25%) when compared to rates for Caucasian teenagers (9%) [4,12].

Evidence suggests that access to care has significantly increased and that the rates of public insurance coverage is greatest among minorities [11]. If this is the case, why do oral health care disparities persist for children in the African American community? There are several social determinants, including race, education, and socioeconomic status, to name a few, which impact healthcare outcomes [3]. Research has shown that race, education, and income all contribute to the disparities and are hard to separate as a higher percentage of African Americans have lower incomes, and education and income are known to be related to health. However, even when education and income are considered, African Americans show increased oral health disparities compared to Caucasians [13]. Nevertheless, the literature often does not address these interactions when considering the effect of race. The primary purpose of this paper is to review the potential factors that perpetuate oral health care disparities in African American children in the United States and describe them in terms of *structural*, *sociocultural*, and/or *familial* factors.

## 2. Methods

A scoping review based on the five-step framework established by Arksey and O’Malley [14] was conducted to identify the scope and nature of existing research regarding the oral health care disparities of African American children.

### 2.1. Step One: Identifying the Research Question

The research question guiding this review was: What are the structural, social, cultural, and/or familial factors that contribute to and/or perpetuate oral health care disparities for African American children?

### 2.2. Step Two: Identifying Relevant Studies

To examine current factors affecting oral care in African American children, research articles published between January 2010 and December 2016 were searched. A search of three databases (PubMed, CINAHL, and ProQuest) was conducted utilizing the terms [(“African American”, “Black”), OR (“Minority Health” and “Health Care Disparities”)] AND (“Oral Health”, “Oral Hygiene”, “Dental Care”). These databases were selected because of their emphasis on qualitative and quantitative research methods and their multidisciplinary approaches. There were large differences in the number of references generated in each database ranging from 32 to 690. In addition to identifying articles through the databases, we reviewed the reference lists of identified articles to ensure that we identified all relevant articles. All research designs were considered, as is typical of scoping reviews.

### 2.3. Step Three: Study Selection

The search of three databases yielded 795 articles. Following the removal of duplicates, 744 articles remained.

These article titles and abstracts were reviewed for relevance. Inclusion in the review required articles to address: (1) oral care in African American children (aged 0–18 years), articles that did not focus solely on but included an African American subgroup were included; and (2) the barriers to oral health (i.e., public or private health insurance). Articles were excluded if: (1) black participants were not African Americans (i.e., black Hispanics from Brazil), (2) oral oncology procedures were the focus of the article, (3) research did not take place in the United States, or (4) articles were written in a language other than English.

Following review of 744 abstracts based on inclusion and exclusion criteria, 33 articles remained. The full articles were obtained and reviewed for appropriateness. Ten articles were excluded following review: four were excluded due to the age of the participants, another four were excluded because the research did not take place in the United States, and two did not include an African American subgroup. A total of 23 articles were included in the review.

### 2.4. Step Four: Charting the Data

Data was charted to aid in the process of identifying relevant information from each article. Articles described oral health problems experienced by African American families and articles assessed efforts to test interventions focused on African American children and their parents.

### 2.5. Step Five: Collating, Summarizing, and Reporting the Results

After reviewing all articles for themes related to the primary question, the authors analyzed the articles using categories informed by the social ecological model (SEM) and Healthy People 2020 [3,15]. The themes—*familial*, *sociocultural*, and *structural* considerations—provided a template for a more holistic understanding of the factors contributing to oral health disparities in African American children.

As reported, the SEM-based themes can be inter-related, with multiple articles addressing issues that may fall in multiple categories. Therefore, the authors of this review have chosen to identify the most tangible contributing category in the descriptive summary and mention overlapping categories when necessary. This information is also reflected in the tables as well as in the Discussion section.

## 3. Results

**Figure 1:**
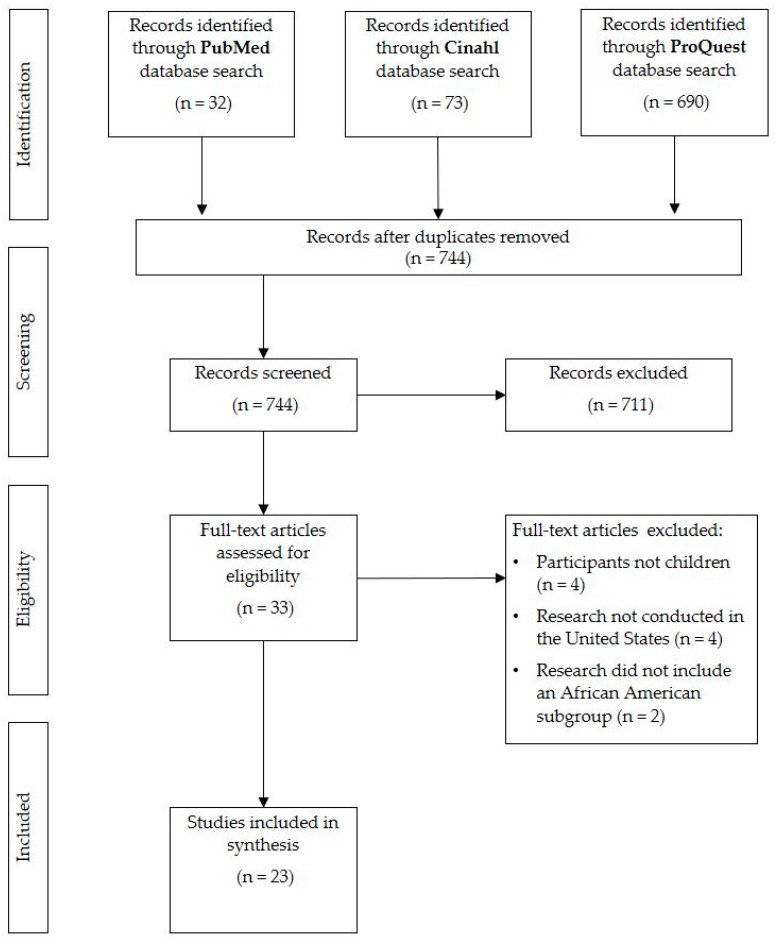
PRISMA flowchart.

**Figure 2:**
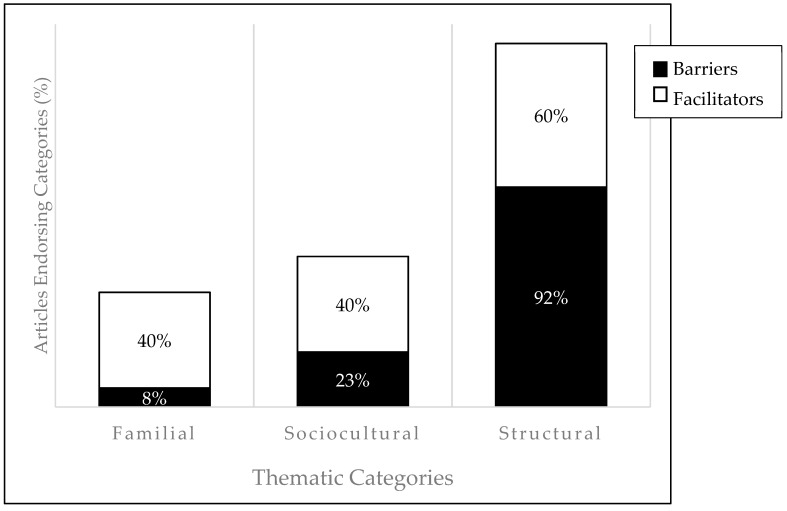
Percentages of review articles’ results categorized into each thematic category. Note: Group percentages do not sum to 100% as articles included in the review could reference more than one thematic category.

A total of 23 articles were included in the review (see Figure 1 for the PRISMA flowchart). Table 1 includes articles that describe the oral health problems African American families encounter by highlighting oral health disparities. Table 2 includes articles that assess efforts to test interventions to improve oral health concerns for African American parents and their children. Both tables include the author(s), date of publication, study purpose, population, study design, and key findings from each study. Percentages of review articles’ results categorized into each thematic category (familial, sociocultural, structural) are visually depicted in Figure 2; divided by barriers (Table 1 results) and facilitators (Table 2 results).

### 3.1. Descriptive Summary

#### 3.1.1. Familial

The familial category is defined as the formal and informal social support systems that can influence individual behaviors from family, friends, and peers [15]. This is critical because of the significant role that parents/caregivers play in their children’s oral care. Research in this category primarily focused on parents’ knowledge and influence on oral health practices. Four of the articles examined parental or caregiver knowledge of preventative measures, attitudes, effective treatment, and insurance coverage policies. One-third of the articles (n = 8) addressed behaviors and practices related to oral care and hygiene through intervention and descriptive observational studies. This included one randomized controlled trial used to evaluate the effectiveness of a tailored intervention on oral health behaviors utilizing motivational interviewing.

In a study to determine early childhood caries prevention treatments utilized by African American parents, results suggested that treatments which allowed for autonomy and a sense of control by either the parent or the child (e.g., tooth brushing versus fluoride) were preferred [29]. Similarly, in the only randomized controlled trial in this review, parents/caregivers participated in an intervention to improve the oral care of their young child (0–5 years); those that received a motivational interview-based intervention were significantly more likely to check for “pre-cavities” and encourage tooth brushing at bedtime at follow up as compared to those who received DVD-based intervention [34]. Additionally, caregivers who utilized routine dental care services for themselves were more likely to bring their children to the dentist for routine care [34]. The previous examples highlight the important role that parent’s knowledge and behaviors provide as supports for the oral health of children.

Parental education level is a known social determinant for the overall health of children. A study reported two significant associations between caregiver education and their child’s oral health: first, lower education of the parent was associated with increased dental caries in the child; second, higher parental education was associated with increased likelihood of utilizing dental services yearly for their child [33]. Higher parental education level was also shown to be a predictor of better oral health for preschool children enrolled in a Florida Head Start program [37].

Lastly, in a participatory action research project conducted with parents of infants and children enrolled in a Women, Infant, and Children (WIC) program in urban Michigan, parents identified many factors that served as barriers to their child’s oral health. Parents stated that time constraints contributed to poor oral health, including limited time available for healthy food preparation and insufficient time off from work to attend routine dental check-ups due to limited hours of operation. They also emphasized that financial constraints negatively impacted healthy food and beverage choices, which resulted in unhealthy food choices for their children [18].

#### 3.1.2. Sociocultural

Sociocultural factors include the social norms, customs, beliefs, knowledge, attitudes, and behaviors that are shared by a population. It is further defined as a collection of people who share a way of thinking, communicating, and behaving specific to the group [15]. The sociocultural theme was utilized rather than separating social and cultural because it is challenging to discern one from the other in the literature. Several articles explored the relationships and associations between social factors that intersect to exacerbate the challenges that many African American families encounter in the pursuit of oral care.

In a study of largely African American (80%) adolescents 12–18-years-old, researchers found that the perceived threat from oral disease was low and that aesthetics were the main reason for seeking care [20]. Aesthetics serve a greater purpose beyond the superficiality it may suggest, providing the confidence required to engage with others and participate in community. For this reason, it is important to consider the social norms of our larger society and the value we place on appearance.

Social development and influence are also of particular importance for adolescents. In a study conducted with youth 11–14-years-old, teens exhibited improvements in at-home oral hygiene behavior following the completion of healthy lifestyle modules led by Morehouse School of Medicine students in a group setting. Modules provided participants with education regarding self-care and opportunities for them to engage in hands-on demonstrations in a developmentally appropriate way. Upon completion of the oral hygiene module, 69% of African American teens improved their oral care behavior (e.g., frequency of tooth brushing and flossing) based on self-reporting [30]. Through engagement and increased knowledge, behaviors of this group of teens positively changed.

With regard to food choices, consumption of sweetened food and less frequent tooth brushing were found to be significantly associated with increased early childhood caries in African American children [32]. African American preschool children who consumed higher amounts of sugar and chips were at increased risk for developing caries [35]. Frequent consumption of cariogenic snacks and non-diet sodas were also shown to significantly increase the odds of experiencing pain in a group of African American adolescents in rural South Carolina [38].

Lastly, an additional sociocultural factor that has been explored as a possible explanation for the continued persistence of oral health disparities is the value placed on patient–provider interactions. African American caregivers report that they are dissatisfied with the oral care their children receive and this dissatisfaction may prevent them from seeking services [23]. Parents of minority children were significantly more likely to say that their health practitioners do not spend enough time with their child, compared to white parents and were more likely to report experiencing unmet oral care needs [22,23].

#### 3.1.3. Structural

The structural category was defined as the rules and regulations for operations that affect how, or how well, services are provided. It also includes institutional networks and the local, state, and federal laws or public policies regarding the allocation of resources and access to health care services [15]. Fifteen articles were sorted into this category.

In a six-year longitudinal study, school-based dental sealant programs were found to decrease barriers to access to dental services for all children, including an African American subgroup [31]. In contrast, utilization of dental services remained low in African American kindergarten children, despite school screenings, referrals, and reminders provided to parents [36].

Based on the literature, geographic location is associated with inconsistent preventative dental service use, particularly for African Americans in rural areas [21,22]. As the authors note, this may be the result of limited access to trained professionals, limited access to transportation to dental clinics, or lack of oral health care as a personal priority. Likewise, older African American children living in rural areas were more likely to have untreated caries, found to be associated with the lack of oral health resources in their immediate environment [19]. Furthermore, the lack of dental clinics in some communities and the scarcity of dental practitioners matriculating through the education pipeline that are willing to work in underserved communities makes participation in preventative dental care a very challenging prospect for rural communities [16,27].

Several articles noted the role of local and national public policies related to access and their impact on overall oral health. For example, researchers found that nearly half of their African American parent participants, in a cross-sectional study, were unaware that their children were eligible for Medicaid/CHIP [24]. Children of families with private insurance showed an increase in preventative procedures until the age of 9 when the utilization of preventative procedures began to decrease. Despite this, African American children received fewer preventative procedures when compared to their Caucasian peers with the same private insurance coverage across all age groups. The authors posited that this change may be the result of complex interactions between race and other factors such as parental perception of oral health need, parental education level, and overall health literacy [17]. Additionally, African Americans with Medicaid were more likely to have longer intervals between dental visits compared to Caucasians with Medicaid [28]. Flores and Lin found that some disparities decreased with improved access to preventive dental services (e.g., completing a routine dental visit in the previous year); however, new disparities, such as greater difficulty accessing specialty services, then emerged for African Americans [24].

Despite the fact that national health policies have significantly reduced the percent of African American children without a dental visit from 1964–2010, socioeconomic status continues to be associated with persistent oral care disparities [25,26]. For example, in studies that were categorized into familial and sociocultural factors, lower socioeconomic status was found to be: (1) a predictor of poor oral health for African American preschool children, (2) associated with greater incidence of oral pain in rural adolescents, and (3) associated with limited food access and unhealthy consumption resulting in increased risks of caries [32,35,37,38].

## 4. Discussion

This scoping review highlights many of the factors that serve as barriers to oral health in the African American community. Persisting disparities in oral health may be why oral health continues as a prominent objective of Healthy People 2020. Structural factors include challenges obtaining access to adequate care, availability of insurance coverage, and the financial cost of services [23,27,28,36]. Although the availability of insurance has increased, research indicates that it remains underutilized by the African American community. For example, public dental insurance coverage has increased from 31% to 62% over the last two decades, most recently attributed to public health efforts to increase children’s dental coverage (i.e., CHIP, Medicare) [26]. However, insurance coverage remains a concern, with African Americans significantly more likely to report being without insurance or, at best, sporadically insured as compared to Caucasians [23]. Older African American children residing in rural areas also share a similar lack of insurance and are more likely to have untreated dental caries compared to children in urban areas [19]. Compounding this issue may be the lack of awareness by some caregivers regarding their child’s eligibility to participate in federally funded insurance programs [24]. Awareness of available resources for underserved communities has also been noted by a group of policy stakeholders as an area to be addressed to improve oral health through health literacy and outreach campaigns [16].

African Americans continue to have higher rates of decay, caries, and missing teeth than many of their peers [4,12,39]. Children with private insurance visited a dentist in the previous year more frequently than those with public insurance and those children without insurance [40]. For those without dental insurance, dental care cost is identified as a major obstacle to care [4,21,38,41]. This is problematic, especially when considering that insurance remains underused.

Sociocultural factors also impact the way African Americans experience oral health, from the patient-provider relationship to the value placed on oral health. African Americans have a tenuous history with the medical community and at times display evidence of mistrust for medical professionals [42,43]. This mistrust can lead to an avoidance of services by African Americans. In a study of African American adults that examined dental fears, patients reported concerns about the poor quality of care they might receive [44]. Siegel and colleagues attributed these concerns to be a direct response to previous experiences with dentists who discounted their feelings of pain and discomfort. Some participants additionally expressed fears that dentists might not adequately clean and sterilize the instruments [44]. It is likely that these sociocultural factors of fear and mistrust have the potential to prevent African Americans from seeking and receiving oral care.

This strained patient–provider relationship may be further exacerbated if the results of a study of dental students’ attitudes about working with underserved populations is any indication of the future of the workforce [45]. The study revealed that the students’ positive attitude about working with underserved populations declined over the course of their dental school training. This could be attributed to many factors, including increased understanding of the challenges that are often present when attempting to address the complex issues that arise when working with underserved populations. Others may argue that the acknowledgement of “real world” expectations (e.g., tuition repayment) might explain negatively sloped, idealistic perspectives.

Stigma has been documented as a barrier to medical (non-dental) health care encounters for minorities, including those with special health care needs [46,47]. Therefore, stigma may also play a role in perpetuating the oral health disparities that persist for African Americans. Although no articles related to stigma were identified in this review, it would be naïve to believe that stigma does play a role in the oral health practices and disparities in African American children. Stigma is a topic that deserves greater attention for this population as it relates to oral health.

Homophily, the tendency to bond with those who are similar, may be a potential facilitator for overcoming patient–provider mistrust and oral health disparities. However, this is not easily achieved for African American patients and practitioners, and may be attributed to the disproportionate growth of minority populations compared to dentists with minority backgrounds. According to a report produced by the American Dental Association, African Americans comprise 12.4% of the population while only 3.8% of dentists self-identified as African American [48]. This, too, may be the result of the historical structural barriers which prevented dentists of color from studying, gaining acceptance to, and participating fully in the field of dentistry, resulting in underrepresented dental practitioners of color and underserved communities of color. In the early 1900s, the formation of a professional organization for people of color was founded to promote dentistry and provide mentorship opportunities, thereby improving the possibility of access to care. This organization would eventually be named the National Dental Association and its goals remain the same today: to improve oral care in underserved populations and education for minority oral health professionals [49].

Socioeconomic status is associated with the oral health of African American children, with an emphasis on the relationship between food and oral health. The consumption of sweetened foods, cariogenic snacks, and sodas are significantly associated with socioeconomic status and access to quality food choices [32,35,38,50]. As a result, the intake of such foods frequently leads to higher rates of caries. Researchers found associations between food insecurity, defined as inadequate access to quality, variety, and desirable food choices, and higher rates of caries in children ages 5–17 [50,51]. Access to high quality food options is a structural factor that may improve oral health for African American children.

As previously stated, many of the SEM-based themes are inter-related. Diet and food choices are highly related to and frequently informed by culture. While structural factors may play a significant role in the ability to access quality food, sociocultural and familial factors may also contribute to the consumption of sugary sweets which negatively impact oral health. In an anthropological study exploring African American identity and food choices for participants with type-II diabetes, the authors found that, “in African American culture, food is the quintessential symbol of love, and… communicate[s] history, memory, feelings, and social status. When no other wealth is available to exchange, food provides both a material and a spiritual form of capital” [52] (p. 163). Expanding beyond the role of food in diabetes studies, consumption of “treats” could be the result of family tradition, used as a way to express love and potentially contributing to damaging oral health practices. These family traditions could also be part of a larger sociocultural norm that is shared by African Americans. Therefore, additional research about the underlying motivations for the consumption of unhealthy foods is needed.

Familial factors are of important consideration for children, in particular, as their care is likely managed by a parent or caregiver. Children’s life circumstances are a direct result of the decisions and opportunities of their parents and caregivers. For example, despite increased access to dental care for children, only 35% of adults 18–44 visit the dentist annually [53]. If parents do not seek dental care for themselves, it is possible that the barriers (e.g., cost, insurance) parents encounter impact their beliefs about oral health practices and thereby influence the oral health practices of their children. As such, it is important to determine what role the family plays in a child’s oral health.

Addressing the oral health needs of adolescents poses its own unique challenges. The articles in this review did not reach consensus about the age range for adolescence although all fell within the range outlined by the World Health Organization for ages 10–19 years [54]. The argument could be made that many in this group are autonomous and generally self-sufficient in their oral care routines; however, with such a large age range, it could also be argued that some are impacted more than others by a combination of structural, sociocultural, and/or familial factors, based upon their age. For example, it is likely that adolescents are more autonomous than younger children in their food choice and consumption of cariogenic snacks and non-diet sodas. Nevertheless, studies examining social norms surrounding healthy food intake for adolescents found that parents continue to play an important role in their selection of healthy foods and their modeling of healthy behaviors being valued more than verbal assertions [55,56].

A possible, and likely, explanation for the persistent barriers to oral health care is that access is not the only reason why African Americans remain underserved. Based on the articles included in this review, the majority of researchers have focused on structural barriers, eschewing potential familial and sociocultural barriers that may exist (see Figure 2). Many aspects must be considered, such as parental income, caregiver education, and geographic location [33,39]. Minimizing the barriers that parents face when seeking oral care for their children should be an area of further exploration. Policymakers should consider the potential benefit of community-based programs on reducing obstacles to access and quality care. For example, the school-based dental sealant program decreased barriers for many participants in Massachusetts, including African American children [31].

Higher educational attainment has shown to be a strong predictor of good oral health across the lifespan for African Americans (34% less untreated tooth decay) and other racial and ethnic categories [33,37]. Overall, lower educational attainment minimizes earning potential and is associated with decreased literacy levels. Lower caregiver literacy is associated with harmful oral health behaviors [57]. Additionally, work-life earning potential is positively correlated with level of education, enabling payment for treatment not covered by insurance and/or co-pays. Lower education, earning potential, and literacy are risk factors for poverty, which heightens the threat to oral health.

An apparent gap in the research of African American children and oral health is the impact of culture on oral health. This is an area that should be examined as culture is identified as a social determinant of health [58]. Additionally, future studies should focus on other minority experiences (e.g., Native American, Alaska native, Latino), replicating this study to examine barriers and facilitators to oral care. This examination of the oral health disparities experienced by other underserved groups will provide valuable information required to influence future intervention development.

### Future Directions

The result of this review identified several structural, sociocultural, and familial factors that impact African American’s ability to utilize oral care services, despite increased access to care. Strategies for how to mitigate these factors need to be explored. Oral care health disparities are often discussed from the perspective of public health, defined as “the science and art of preventing disease, prolonging life and promoting health through the organized efforts and informed choices of society, organizations, public and private communities and individuals” [58]. Health promotion behavior changes are complex. Many influences contribute to the successful implementation of a behavior change, including individual influences, structural, social/cultural, and familial factors [15]. Taking a transactional view of behavior change, clarity begins to form as to why health disparities continue to serve as barriers to the successful enactment of the desired behavior(s). A transactional approach to behavior changes the perspective from the individual as the center of everything to the notion that the contextual factors (e.g., society, environment) also have an impact and that the interactions are fluid and originate in one, or the other, or even both [59]. This an important consideration, as evidence suggests that disparities may persist because a narrow focus on increasing access to care omits several other levels of influence that serve as barriers.

The health care system in the U.S. is also experiencing its own major transition. The Institute for Healthcare Improvement, Triple Aim Initiative is a proposed framework which attempts to improve care by outlining three dimensions that should be addressed simultaneously [60]. These have been identified as the patient experience of care, the health of populations, and the cost of care. Following the assessment of patient experience, holistic treatment has been shown to increase patient satisfaction [59]. As such, practitioners have attempted to find ways to provide more holistic treatment. As dental practitioners explore how to enhance the experiences of their patients, consideration should be given to typically unrecognized factors and utilize a holistic view to move beyond the mouth and concern oneself with the knowledge, attitudes, and beliefs of patients and their families.

## 5. Conclusions

Oral health is an indicator of overall health. Yet for many, oral health care remains an underutilized service. For African Americans in particular, access to oral care has increased as a result of improvements in insurance coverage and through community-based programs; however, disparities persist. A deep dive into the current literature was conducted to parse out additional reasons why these disparities may persist. The literature highlights many of the structural factors that continue to serve as barriers to care for African American families including access to specialty services, an awareness of available public services, and cost-prohibitive pricing. Sociocultural factors identified included the strained patient–provider relationship, possibly the result of historical injustices and workforce limitations, the impact of culture and food, and the value placed on oral care due to social norms. Finally, the familial factors that impact oral health included parental education, parental resources (i.e., time, finances), and parental oral care behaviors. This review also highlighted the fact these factors often are inter-related and somewhat fluid in categorization, with several factors overlapping in multiple categories. The impact of culture on oral health is a factor that deserves greater attention to provide additional insights into the oral health disparities of African American children.

## Figures and Tables

**Table 1:** Articles that describe oral health barriers African American families encounter.

Reference	Purpose	Population	Study Design	Key Findings	Category
**Behrens et al. [16]**	To explore factors and identify strategies that could improve the oral health of low income and minority children	25 state and federal policy makers, workforce experts, foundation officials, educators, researchers with interest in children’s oral health	Qualitative Study; interviews with stakeholder and policy makers	Policy stakeholders believe that improving oral health for children requires addressing: both consumer demand and provider supply, lack of outcry for accessible oral health, undervaluing oral health, health literacy and outreach campaigns	Structural
**Bhagavatula et al. [17]**	To document the rates of prevention, restoration, and surgical dental procedures provided to children enrolled in private insurance, Delta Dental	266,380 children (age 0–18) (12% African American), that received care from 2002–2008 in Milwaukee	Descriptive study; registry summary design	44% of AA had one dental visit during study period; rates of preventative procedures increased to age 9 and then decreased	Structural
**Collins et al. [18]**	To understand what parents consider to be important factors and resources that influence their child’s oral care	Utilized Photovoice with 10 parents of infants and toddlers; five group sessions were conducted	Qualitative Study; participatory research approach	Poor oral health was associated with avoidance of problems; financial constraints, time constraints, and occasional parental frustration completing child’s oral hygiene routines	Familial, Sociocultural, Structural
**Dawkins et al. [19]**	To compare sociodemographic differences between caries and no caries groups and investigate factors associated with untreated dental caries	2453 participants (5.8% African American), children (age 6–15), school-based dental sealant program in KY	Observational Study; pooled cross-sectional design	Older children living in rural areas were more likely to have untreated dental caries and lack insurance	Structural
**Dodd et al. [20]**	To explore oral health perceptions and dental care behaviors among rural adolescents	100 rural youth (age 12–18), (80% Black), low SES	Qualitative study; emergent thematic approach	Perceived threat from oral disease was low, esthetics main reason for seeking care; access, finances, transportation, and fear were also noted	Sociocultural
**Eisen et al. [21]**	Examine relationship between race and dental services	1408 participants (59.3% African American)	Observational Study; cross-sectional analysis of data from The Exploring Health Disparities in Integrated Communities (Baltimore, MD)	More AA used dental services in previous 2 years; place of living an important factor to consider when seeking to understand race difference in dental service use	Structural
**Fisher-Owens et al. [22]**	To assess the extent that factors other than race explain disparities in children’s oral health	Data from National Survey of Children’s Health Children (n = 82,020) (age 2–17)	Observational Study; model based survey data analysis	AA more likely to report poor oral health, lack preventative care, and experience unmet need. However, these are attenuated, to varying degrees, when researchers adjust for socioeconomic status	Structural
**Flores et al. [23]**	To identify racial/ethnic disparities in medical and oral health, access to care, and uses of services in U.S. children	Sample from National Surveys of Children’s Health, parents of 90,117 children (age 0–17), (9.84% African American)	Descriptive study; secondary analysis	Disparities continue to exist, with increased use of services disparities decreased; however, several new disparities for African American children including uninsurance rates and difficulty getting specialty care	Structural
**Flores et al. [24]**	To examine parental awareness of and the reasons for lack of insurance coverage in eligible communities	97 recruitment sites; 267 participants (age 0–18) (35% African American)	Observational Study; cross-sectional design	Half the participants were unaware that their children were eligible for federally funded insurance	Structural
**Guarnizo-Herreño et al. [25]**	To measure inequalities in children’s dental health based on racial/ethnic identity	Representative sample of children and adolescents (age 2–11); White, Black, Hispanic	Observational Study; decomposition model for analysis	SES accounted for 71% of the gap in preventive dental care between AA and White	Structural
**Isong et al. [26]**	To examine the impact of national health policies on AA children’s receipt of dental care	Children 2–17 years old; from 1964 to 2010	Observational study	Percent of AA children without a dental visit declined significantly over time	Sociocultural, Structural
**Lau et al. [27]**	To examine racial/ethnic disparities in medical and oral health status, access to care and use of services in U.S. adolescents	47,728 parent responses from National Surveys of Children’s Health for adolescents (age 10–17), (9.84% African American)	Descriptive study; secondary analysis	Suboptimal health and lack of personal doctor were found to be one of the most profound disparities to exist	Structural
**Pourat et al. [28]**	To look at racial and ethnic differences between children with private insurance and those in Medicaid or CHIP	Sample from the California Survey of Health, 10,805 children (age 0–11) (7% African American)	Descriptive study	AA with Medicare more likely to have longer intervals between visits than Caucasian children with Medicare	Structural

***Note.*** AA = African American; SES = socioeconomic status; CHIP = Children’s Health Insurance Program.

**Table 2:** Studies that describe oral health facilitators and assess efforts to address the oral health problems African American families face.

Reference	Purpose	Population	Study Design	Key Findings	Category
**Adams et al. [29]**	To determine AA parents’ treatment acceptability and treatment preferences to prevent early childhood caries	48 parents/caregivers with an African American child (age 1–5)	Mixed method study; concurrent triangulation design	All treatments were acceptable; parents strongly preferred tooth brushing rather than fluoride varnish and the use of xylitol in gum or food	Familial
**Baker et al. [30]**	Provide developmental program focused on promoting healthy lifestyles to inner-city youth including one module focused on oral hygiene	46 African American youth (age 11–14)	Experimental study; non-randomized controlled trial; 5-week module intervention	Surveys indicated that 42% of the participants exhibited positive behavioral change following completion of the oral hygiene module	Sociocultural
**Devlin et al. [31]**	To evaluate school-based dental sealant programs	Framingham school district 2^nd^ graders (≈6% African American) with dental sealants	Experimental; non-randomized controlled trial	School based dental sealant programs can help decrease barriers for access to dental services	Structural
**Ghazal et al. [32]**	To assess the relationship between behavioral factors and caries in AA preschoolers	96 African American children (age 3–22 months)	Observational study; longitudinal cohort study	Living in a non-fluoridated community, more frequent consumption of sweetened food, less frequent consumption of 100% juice, less frequent tooth brushing, significantly associated with greater ECC incidence.	Sociocultural, Structural
**Heima et al. [33]**	To investigate the influence of caregiver education level on dental caries	423 children (age 5–6) and caregiver dyads (94% African American), low income, urban	Cross-sectional design; secondary analysis of longitudinal study data	Caregiver education level was associated with 34% less untreated decayed teeth	Familial
**Ismail et al. [34]**	To evaluate the effectiveness of a tailored intervention on oral health behaviors and new untreated caries	1021 randomly selected African American children (age 0–5) and their caregivers	Experimental study; randomized controlled trial	Caregivers receiving motivational interviewing and watching DVD more likely to report checking for “pre-cavities”	Familial
**Johansson et al. [35]**	To investigate the association between snacking and caries	1206 preschool children (age 1–4) (61% African American)	Observational study; cross-sectional design	Presence of plaque, sugar intake and SES were associated; consumption of chips was associated with caries	Sociocultural, Structural
**Nelson et al. [36]**	To assess follow-up dental care received by children given baseline screening and referrals as part of an ongoing clinical trial	303 participants (age 5–6), (96% African American), who had at least one dental visit	Observational study; retrospective cohort design	Utilization of dental services was low for poor minority inner city kindergarten children despite school screening referrals and parental reminders	Structural
**Weatherwax et al. [37]**	To identify possible relationships between parent/guardian sociodemographic, intention, knowledge, and oral health status of their child	181 child (age 3–5) and parent/ caregiver dyad (31% African American), Head Start program	Observational study; cross-sectional design	Caregiver race/ethnicity and years of education were inversely significantly associated with decayed, missing or filled teeth	Familial, Structural
**Yuen et al. [38]**	To explore behavioral factors associated with toothaches among African American adolescents	Convenience sample of 156 African American adolescents (age 10–18)	Observational study; cross-sectional design	Age and consumption of cariogenic snacks and soda are related to toothache pain	Sociocultural, Structural

***Note.*** ECC = early childhood caries.
